# The Host-Pathogen Interactions and Epicellular Lifestyle of *Neisseria meningitidis*

**DOI:** 10.3389/fcimb.2022.862935

**Published:** 2022-04-22

**Authors:** August Mikucki, Nicolie R. McCluskey, Charlene M. Kahler

**Affiliations:** ^1^ Marshall Centre for Infectious Diseases Research and Training, School of Biomedical Sciences, University of Western Australia, Perth, WA, Australia; ^2^ College of Science, Health, Engineering and Education, Telethon Kids Institute, Murdoch University, Perth, WA, Australia

**Keywords:** invasive meningococcal disease, pathogenesis, microbiome, inflammation, evolution

## Abstract

*Neisseria meningitidis* is a gram-negative diplococcus and a transient commensal of the human nasopharynx. It shares and competes for this niche with a number of other *Neisseria* species including *N. lactamica, N. cinerea* and *N. mucosa*. Unlike these other members of the genus, *N. meningitidis* may become invasive, crossing the epithelium of the nasopharynx and entering the bloodstream, where it rapidly proliferates causing a syndrome known as Invasive Meningococcal Disease (IMD). IMD progresses rapidly to cause septic shock and meningitis and is often fatal despite aggressive antibiotic therapy. While many of the ways in which meningococci survive in the host environment have been well studied, recent insights into the interactions between *N. meningitidis* and the epithelial, serum, and endothelial environments have expanded our understanding of how IMD develops. This review seeks to incorporate recent work into the established model of pathogenesis. In particular, we focus on the competition that *N. meningitidis* faces in the nasopharynx from other *Neisseria* species, and how the genetic diversity of the meningococcus contributes to the wide range of inflammatory and pathogenic potentials observed among different lineages.

## 1 Natural History of the meningococcus

### 1.1 Epidemiology

*Neisseria meningitidis* (Nme) is an obligate coloniser of the human nasopharynx and upon invasion of the host, can cause invasive meningococcal disease (IMD). There are two primary clinical manifestations of IMD: meningococcemia (20-30% of cases), which presents as a petechial or purpuric rash, and meningitis (50-60% of cases); characterized by fever, vomiting, headache, photophobia, agitation, drowsiness, and stiffness of the neck (Pace and Pollard, 2012). IMD progresses rapidly with high mortality (4.1%-20.0%) despite intensive treatment with β-lactam antibiotics including penicillin and ceftriaxone (Wang et al., 2019). Among survivors of IMD, 20% will experience long-term morbidity. Sequelae include neurological/hearing impairment, chronic pain, scarring, and amputation following septicaemia; while seizures, visual impairment and motor deficits are characteristic of meningitis (Pace and Pollard, 2012). In rarer instances, Nme may cause atypical disease presentations including meningococcal arthritis, pericarditis, pneumonia, and urethritis (Vienne et al., 2003; Bazan et al., 2017). Some reports also indicate that Nme may induce meningitis *via* infection of the olfactory nerve, bypassing the bloodstream and leading to meningitis in the absence of bacteraemia (Sjölinder and Jonsson, 2010; Delbaz et al., 2020).

Transmission of Nme between people occurs *via* large respiratory droplets spread by direct inhalation to other individuals in close proximity, although minor modes of transmission *via* the urogenital and anorectal secretions have recently been detected (Ladhani et al., 2020). It is unclear whether fomites play a role in transmission. However, under laboratory conditions the meningococcus can survive on surfaces for up to a day (Swain and Martin, 2007). Upon acquisition, human organoid models have shown meningococci preferentially bind to the microvillous surface of non-ciliated cells of the human nasopharynx, located at the back of the nose and above the oropharynx (Stephens et al., 1983). After initial contact, the bacteria form microcolonies which stably colonise the epithelial surface. Carriage of a single meningococcal isolate may persist for 5-6 months before clearance, depending on the host and isolate in question (Caugant and Maiden, 2009). The mechanism by which meningococci are cleared from the nasopharynx is unknown but includes the induction of natural immunity (Pollard and Frasch, 2001). Carriage prevalence varies by age, peaking at approximately 20% in the 15–20-year-old age bracket before gradually declining in later adulthood (Christensen et al., 2010). The greatest risk factors for Nme carriage are age, high density living situations such as those observed in military and university accommodation and at mass gatherings (Peterson et al., 2018), sore throat, season (Cooper et al., 2019), and behaviours including smoking, alcohol consumption, nightclub attendance, and having multiple kissing partners (MacLennan et al., 2021). In the meningitis belt of sub-Saharan Africa, arid conditions experienced during the dry season significantly increases the risk of meningococcal carriage (Cooper et al., 2019). Viral infection, particularly with Influeza virus A, also predisposes an individual to carriage (Tuite, et al., 2010).

The prevalence of IMD is correlated with meningococcal carriage in adolescents, who drive transmission in the wider population due to increased participation in risk-behaviours (MacLennan et al., 2021). The prevalence of IMD also fluctuates within populations and between geographic regions. Endemic disease (age standardised rate <10/100,000 population/year) is sporadic IMD caused by un-related strains as they circulate in the population (Jafri et al., 2013). Epidemics (age standardised rate >100/100,000 population/year) typically occur upon the introduction of a strain that is antigenically distinct from local carriage isolates (Jafri et al., 2013). This manifests as outbreaks characterized by transmission networks of close contacts with IMD stemming from infection by the same strain, or as waves of hyper-endemicity in which increased incidences of IMD may last for a decade or more in a given area (Holmes et al., 2020).

Strains are typed according to two schemes: into serogroups based on capsular polysaccharide composition (Harrison et al., 2013) and into sequence types (ST) based on the alleles of seven housekeeping genes using multi-locus sequence typing (MLST) (Maiden et al., 1998). Lineages with STs sharing four or more alleles are grouped into a single clonal complex (cc). Of the twelve known serogroups (A, B, C, E, H, I, K, L, W, X, Y, and Z), six (A, B, C, W, X, and Y) are associated with strains causing the majority of IMD outbreaks (Acevedo et al., 2019). Certain clonal complexes are also associated with epidemics and outbreaks on a global scale. These lineages, of which there are eleven, have been termed the hyperinvasive lineages (Caugant and Maiden, 2009). For example, serogroup A isolates from cc5 (MenA:cc5) were the cause of disease in the African meningitis belt from 1988 to 2001 (Nicolas et al., 2005; Caugant and Brynildsrud, 2020), MenB:cc32 caused outbreaks in the UK during the 1980s (Abbott et al., 1985), and MenB:cc41/44 was responsible for outbreaks in New Zealand, 1990-2005 (Oster et al., 2005). More recently, MenW:cc11 has been the cause of a global outbreak which began during the Hajj pilgrimage in 2000 (Taha et al., 2000) leading to subsequent epidemics in the African meningitis belt and increased outbreaks in South Africa, South America, Europe, and Australia (Caugant and Brynildsrud, 2020). These MenW:cc11 strains resulted from a capsule switching event in which the genes for the synthesis of a serogroup W capsule were acquired by an ancestral MenC:cc11 isolate following a sequence of homologous recombination events (Mustapha et al., 2016). Capsule switching events represent a potential mechanism by which meningococcal lineages may evade vaccine-derived immunity against capsule (Lucidarme et al., 2017).

### 1.2 Genetic Diversity of *N. meningitidis*

Large natural history population-based surveys of meningococcal carriage show that strains belonging to certain clonal complexes are over-represented in IMD versus carriage (Caugant and Maiden, 2009). The IMD disease/carriage ratio (D/C ratio) is used to stratify clonal complexes by their propensity to cause disease. The eleven hyperinvasive lineages, which have been responsible for the majority of IMD epidemics, were found to have an increased D/C ratio compared to other lineages (Caugant and Maiden, 2009). Although the D/C ratio is an observational metric of the association of a genetic lineage with IMD in humans, it demonstrates that lineages differ in their ability to colonise the host and cause invasive disease. This hypothesis was modelled mathematically by Stollenwerk et al. (2004) who predicted that differences in the metabolism and virulence of meningococcal clonal complexes could explain these observations. Studies examining small genome datasets suggest that Nme has a large common array of genomic islands, but that these are present in unique combinations in each clonal complex (Snyder et al., 2001; Stabler et al., 2005; Hotopp et al., 2006; Schoen et al., 2008; Marri et al., 2010). While a subset of these genomic islands has confirmed roles in virulence, the majority have unknown functions or are proposed to have roles in metabolism. Schoen et al. (2014) proposed a model of nutritional virulence in which differences in key metabolic pathways in each clonal complex contributes to niche adaptation. They identified lactate metabolism, the oxidative stress response, glutathione metabolism and the denitrification pathway as key indicators of the involvement of metabolism in virulence. Lactate is generated by anaerobic glycolysis in host cells in response to stress to the extent that during bacterial meningitis, lactate concentrations rise to 13.6 mM, almost 7-fold above the levels in healthy tissue (Llibre et al., 2021). This acts as a carbon source to accelerate bacterial growth. Meningococcal carriage isolates that are not associated with IMD are genetically diverse and distinct from the hyperinvasive lineages. Comparisons of their transcriptional responses to growth in blood, saliva and CSF by Ampattu et al. (2017) have shown that although these isolates retain genetic similarity to invasive isolates, regulation of the pathways involved in energy, glutamine, and cysteine metabolism are quite distinct.

**Figure 1:**
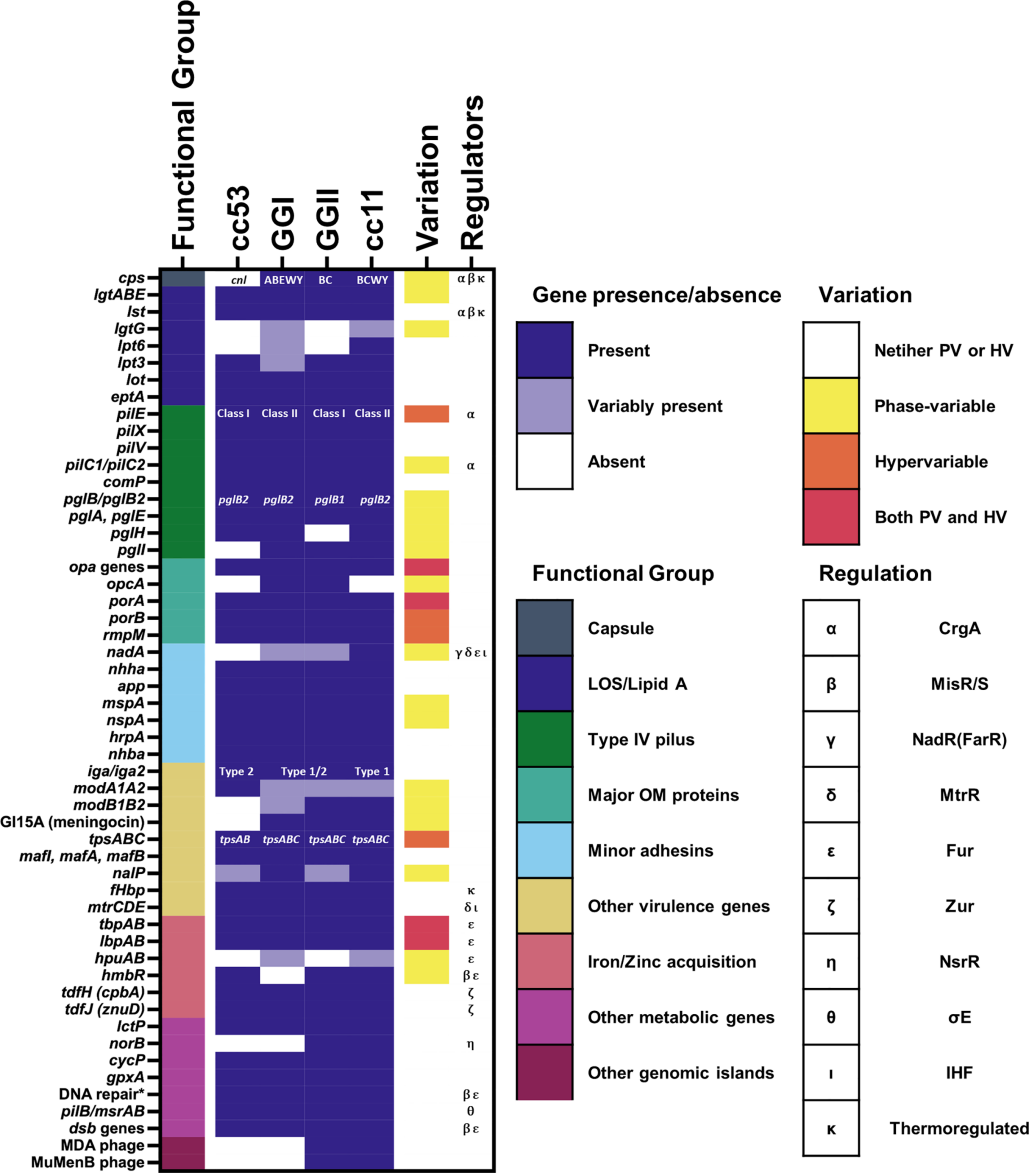
Presence of known genes associated with pathogenesis among meningococcal lineages. The presence of genes associated with pathogenesis mentioned in this review are marked as either present (gene is present in >80% of isolates from the lineage), variably present (gene is present in 20-80% of the isolates from the lineage), or absent (gene is present in <20% of isolates from the lineage). For references see text. Lineages here are defined according to the framework of Mullally et al., 2021. Where specific variants of a virulence gene/island are known, i.e. for capsular serogroup, class of *pilE*, *pglB* allele, and IgA protease cleavage type, the variant predominantly associated with each lineage is indicated. The phase-variation status and known regulators of each gene are also indicated. The known regulators of each gene and the presence of phase-variation and hypervariability are also indicated. * = genes encoding DNA repair enzymes with a known role in resistance to oxidative and nitrostative stress including: *nexo*, *nape*, *nth*, *mutM*, and *dinG*.

A recent study of approximately 4000 genomes of both hyperinvasive and non-virulent genetic lineages by Mullally et al. (2021) identified a cohort of 93 genomic islands with associations across nine hyperinvasive lineages and one non-virulent lineage (cc53). When clustered by the presence or absence of these islands, the hyperinvasive lineages fell into two large but distinct groups, termed genogroup I (GGI) and genogroup II (GGII) (**Figure 1**). Under this scheme, the possession of genomic islands was correlated with the D/C ratio, with GGI (cc5, cc22, cc23, and cc60) possessing fewer genomic islands and a D/C ratio < 0.5 and GGII (cc32, cc41/44, cc213, cc269, and cc461) possessing more genomic islands and a D/C ratio > 0.5.

## 2 Acquisition

### 2.1 Meningococcal Interaction With Mucosal Host Defenses

**Figure 2:**
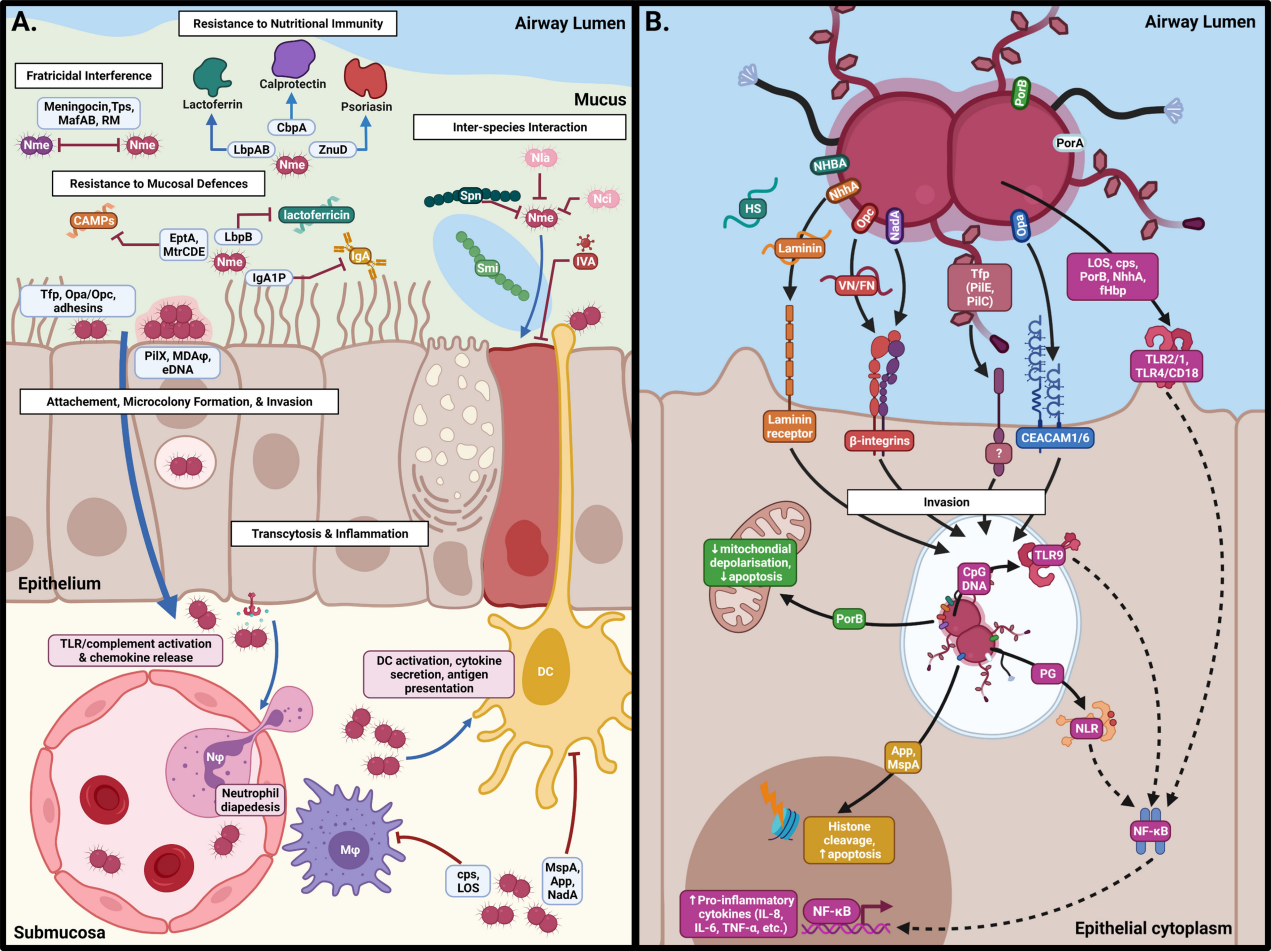
Interactions of *N. meningitidis* at the nasopharyngeal surface. **(A)** Following acquisition, Nme must survive against host defences, interactions with other members of the microbiome, and intra-specific competition in order to inhabit the mucus of the nasopharynx. Eventual binding of the epithelial surface results in intimate association and passage into the submucosa by transcytosis. **(B)** Binding of epithelial cells occurs by interaction of Neisserial surface structures with their cognate receptors, resulting in inflammation, cellular restructuring, and transcytosis. This figure was created using Biorender.com.

The nasopharyngeal respiratory epithelium is covered by a 10-12 µm thick two-layer surface liquid composed of a low viscosity periciliary liquid and a high viscosity mucus that faces the lumen (Lillehoj et al., 2013). The low viscosity periciliary liquid facilitates ciliary beating which continually transports mucus from the lower respiratory tract to the pharynx where it is swallowed, to remove microorganisms and other debris. Mucins are a diverse family of high molecular weight, heavily glycosylated proteins which are secreted into the periciliary fluid or are anchored to the epithelial surface to capture microbes to prevent access to the host cell surface (Derrien et al., 2010). Models of meningococcal colonisation of the nasopharynx had presumed that the meningococcus would make direct contact with the epithelium (Virji, 2009). However, Audry et al. (2019) showed using an air interface culture (AIC) model that the likely niche of Nme in the carriage state is within the mucus layer rather than on the epithelial cell surface, as it lacks swimming motility and mucin-degrading enzymes found in other bacteria. The position of Nme within the mucosal secretions of the epithelium likely reflects a need for protection against desiccation while providing access to nutrients (Audry et al., 2019). Eventual clearance of Nme from the mucosal layer is mediated by the actions of various host defences including secretory IgA (sIgA) (Brandtzaeg, 2013), cationic antimicrobial proteins (CAMPs) (Ganz, 2002) and nutrient restriction (**Figure 2A**).

Human sIgA is the most abundant antibody class at mucosal surfaces and inhibits microbial-host adhesion by non-specifically coating the bacterial surface, resulting in enhanced opsonophagocytosis by IgA receptor-bearing phagocytes (Brandtzaeg, 2013). To counteract human sIgA, Nme expresses an IgA1 protease that cleaves the exposed hinge region of sIgA1 (Mulks et al., 1980). Cleavage at these sites results in the separation of the two antigen-binding Fab fragments from the Fc tail. Cleaved Fab fragments retain antigen-binding activity and bind to surface epitopes on Nme, competitively inhibiting bactericidal antibody binding (Mansa and Kilian, 1986). Nme IgA1 proteases can be divided into two classes (type 1 and type 2) based on their cleavage specificities (Mulks et al., 1980). IgA1 protease cleavage type 1 has the additional capacity to degrade IgG3, which are typically bactericidal antibodies that activate opsonophagocytosis (Spoerry et al., 2021). Interestingly, cc11 isolates exclusively encode cleavage type I IgA1 proteases (Spoerry et al., 2021).

CAMPs are a class of short peptides secreted by the host cells which bind negatively charged bacterial surfaces, disrupting membrane integrity and leading to bacterial lysis (McCormick and Weinberg, 2010). CAMPs are very diverse in function and origin and not all forms of CAMPs have been tested against Nme. Binding of CAMPs to gram-negative bacteria relies on the overall negative charge of the bacterial surface, which is conferred by the phosphorylated headgroups of the lipid A molecules making up 70% of the outer membrane. In Nme, this negative charge can be ablated by the substitution of the lipid A headgroups with phosphoethanolamine (PEA) by the lipid A ethanolamine transferase, EptA (formerly LptA) (Cox et al., 2003; Kahler et al, 2018). Although EptA is considered the primary mechanism in Nme conferring resistance to CAMPs, there are secondary systems of resistance (Tzeng et al., 2005). Capsular polysaccharide and binding of human factor H by factor H-binding protein (fHbp) on the bacterial surface interferes with the electrostatic interactions between CAMPs and lipid A, thus reducing their effectiveness (Spinosa et al., 2007; Seib et al., 2009). Mutation in the pilin apparatus reduces the influx of CAMPs and the multiple resistance efflux pump, encoded by the *mtrCDE* operon, effluxes CAMPs (Tzeng et al., 2005). In *N. gonorrhoeae*, the MtrR repressor and MtrA activator are responsible for regulation of the *mtrCDE* operon. However, in Nme the insertion of a Correia element into the promoter has resulted in the loss of regulation by this system. Instead, the Correia element contains an integration host factor (IHF) binding site, and repression of *mtrCDE* expression is carried out *via* binding of IHF and post-transcriptional cleavage of the Correia element (Rouquette-Loughlin et al., 2004). MtrCDE efflux pump and capsule expression are induced *via* an unknown mechanism in the presence of sub-lethal concentrations of cathelicidin LL-37 and protegrin-1 (Spinosa et al., 2007), and both EptA expression and capsule synthesis is regulated by the two-component system MisRS (Tzeng et al., 2008; Bartley et al., 2013). Shedding of the outer membrane as blebs also removes CAMPs bound to the bacterial outer membrane (Tzeng and Stephens, 2015). Additionally, the secretion of extracellular DNA (eDNA) may bind CAMPs reducing the effective concentration of CAMPs on the bacterial cell surface (Wassing et al., 2021).

At mucosal surfaces, iron and zinc, which are required for bacterial growth, are sequestered by an array of human proteins, supressing bacterial growth in these environments in a process termed nutritional immunity (Cornelissen, 2018). Iron is sequestered and transported throughout the body by a variety of carrier proteins including transferrin, haemoglobin and haptoglobin. Lactoferrin is the primary protein secreted into mucosal surfaces to sequester free iron, thus restricting the growth of microbes in this compartment (Kell et al., 2020). Lactoferrin binding protein A (LbpA) binds holo-lactoferrin, extracting bound iron and releasing apo-lactoferrin. The LbpB lipoprotein acts in concert with LbpA, enhancing the ability of LbpA to bind lactoferrin. Additionally, lactoferrin is proteolytically processed by host enzymes to release a 47 amino acid peptide, lactoferricin, which acts as a CAMP (Gifford et al., 2005). Resistance of Nme to lactoferricin is conferred by binding of lactoferricin by two negatively charged repeat-regions of LbpB (Morgenthau et al., 2014). Zinc is similarly sequestered by calprotectin and psoriasin and Nme expresses receptors, CbpA (TdfH in *N. gonorrhoeae*) and ZnuD (TdfJ in *N. gonorrhoeae*), which respectively bind these proteins in order to acquire host zinc (Maurakis et al., 2019). Nme does not express its own siderophores but does have the capability of utilising xenosiderophores of other species *via* the FetABC transporter (Cornelissen, 2018).

### 2.2 Synergism and Antagonism With the Human Nasopharyngeal Microbiome

The mucus layer is colonised by a microbial community that may have both synergistic and antagonistic interactions with Nme. During early life, stable microbial communities are established which are dominated by one of six bacterial genera- *Moraxella, Streptococcus, Corynebacterium*, *Staphylococcus, Haemophilus* and *Alloiococcus*. (Durack and Christophersen, 2020). In these situations, *Neisseria* spp. are transient, low abundance members of these communities. Both antagonistic and synergistic interactions have been noted between *Streptococcus* spp. and Nme. *S. pneumoniae* can successfully limit and eliminate competitive flora in co-culture experiments *via* the production of hydrogen peroxide, which is bactericidal against Nme despite Nme possessing catalase activity (Pericone et al., 2000). Additionally, *S. pneumoniae* produces a neuraminidase which desialylates the lipopolysaccharide (LOS), sensitising Nme to complement-mediated killing (Shakhnovich et al., 2002). Conversely, direct synergism between Nme and *S. mitis* has been observed by Audry et al. (2019). Using the AIC model, they showed that *S. mitis* degrades mucins, enabling Nme to reach the epithelial surface, initiating stable colonisation and potentiating growth. This result seems somewhat paradoxical given that both *S. mitis* and *S. pneumoniae* produce H_2_O_2_ by the action of the pyruvate oxidase SpxB (Redanz et al., 2018). Such discrepancies may be due to Nme strain variation in H_2_O_2_ sensitivity, variable amount of H_2_O_2_ expressed by the Streptococcal isolates, or the fact that the study by Pericone et al. (2000) was performed in rich media co-culture, in the absence of host cells and mucus. Infection with respiratory viruses, including respiratory syncytial virus (RSV) and Influenza virus A has also been correlated with increased risk of IMD (Cartwright et al., 1991; Brundage, 2006; Jacobs et al., 2014; Salomon et al., 2020). Infection modelling suggests that the neuraminidase of Influenza virus A can degrade the bacterial sialic acid capsule and therefore enhance the adhesion of Nme to the host epithelium (Rameix-Welti et al., 2009). Dysregulation of the host immune system also plays a role in increasing susceptibility to IMD in a mouse model of co-infection (Alonso et al., 2003). The evidence supporting a correlation between RSV and an increased risk of IMD is conflicting. Tuite et al. (2010) found an association between RSV and IMD, while Stuart et al. (1996) and Jacobs et al. (2014) did not.

Apart from Nme, there are at least nine other commensal *Neisseria* spp. which inhabit the nasopharyngeal mucosa (Liu et al., 2015; Diallo et al., 2019). Colonisation with *N. lactamica* is inversely correlated with the carriage of Nme in humans. *N. lactamica* is the dominant *Neisseria* species during early life, but is replaced by Nme following one year of age (Cartwright et al., 1987). A pharyngeal carriage study from Africa examining six *Neisseria* spp. did not detect any relationships between Nme and the other five *Neisseria* spp. in their test panel (Diallo et al., 2016). In laboratory models of infection, *N. lactamica* and *N. cinerea* measurably inhibit colonisation of immortalised epithelial cells by Nme (Evans et al., 2011; Deasy et al., 2015; Wörmann et al., 2016). It is hypothesised that *N. lactamica* reduces meningococcal carriage both by competitive displacement of resident Nme and by preventing further acquisition (Deasy et al., 2015). *N. cinerea* adheres to the epithelium and forms microcolonies with close associations in a similar manner to Nme (Wörmann et al., 2016). *N. cinerea* significantly impairs meningococcal-host association, microcolony formation and microcolony expansion on host epithelial cells (Custodio et al., 2020). This was attributed to a reduction in meningococcal motility by an unknown mechanism (Custodio et al., 2020).

Two mechanisms of interference between *Neisseria* spp. are mediated by Type IV and Type VI systems. Kim et al. (2019) showed all commensal *Neisseria* spp. can kill the pathogenic *Neisseria* spp. through a mechanism based on competence and DNA methylation. Nme is transformable and possesses multiple restriction modification systems designed to methylate the host chromosome while degrading foreign DNA (Budroni et al., 2011). Kim et al. (2019) found that high concentrations of foreign DNA from the commensal *Neisseria* spp. can overcome this protective mechanism, resulting in the recombination of under-methylated sequences into the host genome, which are subsequently nicked by host restriction endonucleases resulting in the abortion of cell division. It is currently unclear whether this type of inference by commensal *Neisseria* spp. occurs between lineages of Nme, however, the diversity and lineage-restricted nature of restriction modification systems in Nme suggests this may also be a mechanism for fratricidal killing (Claus et al., 2000; Srikhanta et al., 2009; Srikhanta et al., 2010; Budroni et al., 2011). Type VI secretion systems have been identified in multiple commensal *Neisseria* spp. (Calder et al., 2020; Custodio et al., 2021). Custodio et al. (2021) demonstrated in competition assays using *N. cinerea* that the Type VI secretion system resulted in a 50- 100-fold reduction in wild-type survival of Nme. The expression of the polysaccharide capsule enhanced meningococcal survival, and the mechanism of susceptibility required the expression of Type IV pili (Tfp) which is necessary for competence (Custodio et al., 2021).

Although carriage studies rarely report co-colonisation of meningococcal isolates, longitudinal studies of meningococcal asymptomatic carriage revealed the exchange of strains in the nasopharyngeal compartment over time (Barnes et al., 2017), implying that there are fratricidal mechanisms of competition between Nme strains. Two mechanisms have been examined: secreted bacteriocins and contact-dependent killing mechanisms mediated by the TspABI and MafABI systems. Allunans et al. (2008) identified an Nme isolate that inhibited the growth of other strains of Nme on solid media. They showed that this effect was due to the secretion of a bacteriocin (aka meningocin), encoded on a genetic island termed IHT-A2. Some lineages possess IHT-A2 but have non-functional genes for secretion of the bacteriocin (Mullally et al., 2021), suggesting that these strains may retain immunity to killing (Allunans et al., 2008). The TpsBAI(C) and MafABI systems are unrelated two-partner secretion systems that secrete a polymorphic toxin component, TpsA and MafB, which enter strains that are not expressing the cognate immunity factor (TpsI and MafI) resulting in cell death (Tommassen and Arenas, 2017). TpsB mediates the secretion of a cognate TpsA protein (Arenas et al., 2013b) while it appears that MafB secretion is independent of MafA. In some strains, the *tps* island contains short repeating cassettes downstream of the *tpsI* gene, termed *tpsC*, which are proposed to enable recombination with variable 3’ end of *tpsA* to generate new variants of the TpsA toxin (Arenas et al., 2013b). Overexpression of one of the four MafB toxins of strain NEM8013 provided an advantage in competition assays, suggesting a role in niche adaptation (Jamet et al., 2015). The possession of the *tspC* array and the Maf system are characteristic of the hyperinvasive lineages but not the commensal Nme lineage cc53 (Mullally et al., 2021). However, cc53 lineage encodes a potential secreted bacteriocin suggesting that this lineage has evolved to possess fratricidal mechanisms distinct from the hyperinvasive lineages.

## 3 Colonization of the Nasopharyngeal Epithelium

Following the acquisition of Nme by the host, meningococci must undergo several discreet phases of colonisation to become invasive and cause IMD. These are long distance attachment by Tfp, re-traction of the pilus, stable colonisation and microcolony formation, and intimate adhesion to the epithelial surface. During intimate adhesion, meningococcal adhesins initiate remodelling of epithelial cell architecture, resulting in engulfment and transcytosis of Nme to the sub-epithelial layers (**Figure 2B**).

### 3.1 Initial Attachment by the Type IV Pilus

The Tfp is a long filamentous structure composed of the pilin monomer PilE and minor pilins ComP, PilV, and PilX (Carbonnelle et al., 2009). Pilus biogenesis is a complex process involving over 20 different proteins, ultimately resulting in the assembly of pilin polymers in the cytoplasm and extrusion through the outer membrane by PilQ (Carbonnelle et al., 2006; Brown et al., 2010). The retraction of pili is mediated by PilT and is counterbalanced by several proteins, including PilX, PilV, and the pilus-associated adhesin PilC, which regulate the number of pili per bacterial cell (Imhaus and Duménil, 2014). Piliation is required for self-aggregation, adhesion to host cells, and signalling to host cells, respectively (Imhaus and Duménil, 2014). Initial adhesion of Tfp to epithelial cells is mediated by the tip-adhesin, PilC, and along the shaft *via* PilE (Kennouche et al., 2019). Meningococci express two forms of PilC, PilC1 and PilC2, which are regulated independently of one another and modulate pilus function (Morand et al., 2009). While both forms of PilC can mediate adhesion to epithelial cells and induce the formation of cortical plaques, PilC1-based adhesion results in a sharp reduction in the expression of endothelial growth factor receptor (EGFR), which signals epithelial cells to detach from the substratum (Morand et al., 2009). This suggests that the variant forms of PilC allow meningococci to fine-tune host cell behaviour during infection. The search for the receptor for Tfp on epithelial cells has been inconclusive. Early studies identified membrane cofactor protein, also known as CD46, as the Tfp receptor on epithelial cells (Källström et al., 1997). CD46 is a transmembrane glycoprotein found abundantly on nearly all human cells/tissues, including cells of the respiratory tract. However, the role of CD46 in meningococcal adhesion has been challenged by several studies, leaving the identity of the cognate receptor for Tfp at the epithelial surface in question (Tobiason and Seifert, 2001; Johansson et al., 2003; Kirchner et al., 2005; Sutherland et al., 2010).

Pili are post-translationally modified with glycans, phosphocholine, phosphoethanolamine or phosphoglycerol (Bartley and Kahler, 2014; Mubaiwa et al., 2017). Glycans can be di- or tri-saccharides with are variably decorated with *O*-acetyl groups. These glycans are O-linked galactose (α1-3)-*N*,*N*’-diacetylbacillosamine (Gal-diNAcBac) or tri-saccharides of Gal(β-1-4)Gal-diNAcBac or Gal(β-1-4)Gal-GATDH (glyceroamido acetamido trideoxyhexose) (Bartley and Kahler, 2014). Pilin glycosylation may slightly alter pili density and modulate epithelial attachment (Virji et al., 1993b; Marceau et al., 1998). Pilin glycosylation and phosphorylcholine modifications on pilin have been shown to be necessary for interaction with platelet activating factor receptor (PAFr), a key early receptor of the interaction between Nme and host bronchial epithelial cells (Jen et al., 2013). In *N. gonorrhoeae*, the pilin glycan is also essential for the interaction of the pilus with the I-domain of the CR3 receptor, a key mediator of attachment of *N. gonorrhoeae* to primary human cervical epithelial cells (Jennings et al., 2011) and presumptively has a similar role for Nme attachment to CR3 expressing host cells.

### 3.2 Microcolony Formation

Following Tfp mediated attachment, meningococci form bacterial aggregates on the apical surface of epithelium termed microcolonies (Hélaine et al., 2005). Microcolony formation increases attachment at the epithelial surface and allows meningococci to weakly resist shear stress generated by mucociliary flow (Lécuyer et al., 2012). Microcolony aggregation is dependent upon the minor pilins PilX, PilV, and PamA (Pilus associated molecule A), which are required for twitching motility (Imhaus and Duménil, 2014; Takahashi et al., 2020). Microcolonies may progress in two ways: they may evolve into biofilms that result in stable colonisation of the epithelium, or they may disperse. The formation of a biofilm is a trait associated with Nme isolates that have lost the capacity to express capsule (Lappann and Vogel, 2010). Phase-variation of the polysialyltransferase of serogroup B strains, insertion of mobile genetic elements into the promoter of capsule synthesis genes, the transcriptional regulator CrgA, the MisRS two-component system, and temperature have all been shown to play a role in regulating capsule expression (Loh et al., 2013; Tzeng et al., 2016). eDNA is a major component of meningococcal biofilms, and microarrays examining gene expression in microcolonies identified increases in expression of the membrane-bound lytic transglycosylases A and B (MltA/B), which are necessary for autolysis and the release of eDNA (Lappann and Vogel, 2010). Some lineages, including the hyperinvasive lineages cc11 and cc8, form eDNA-independent biofilms. Instead, cc11 possesses multiple copies of the prophage designated MDAΦ (meningococcal disease associated island) (Bille et al., 2005; Bille et al., 2008), which encodes a functional filamentous phage. The MDAΦ phage aids microcolony formation by stabilising inter-bacterial interactions through the formation of phage bundles that extend from the bacterial surface (Bille et al., 2017). These bacteria-bacteria interactions increase the overall biomass of encapsulated Nme interacting with the host epithelium, leading to an increased bacterial load at the site of attachment which in turn enhances the likelihood of bacterial translocation into the bloodstream (Bille et al., 2017). Dispersal of the biofilm is necessary for transmission and two mechanisms have been proposed: a host signal in the form of lactate which is a signal for increased inflammation (Sigurlásdóttir et al., 2017) and the induction of PilE phosphoglycerol transferase B (PptB), which decorates the surface proteins thus changing the dynamics of the bacterial-bacterial interactions (Chamot-Rooke et al., 2011).

Aggregation and biofilm formation by Nme is supported by the minor adhesins: IgA1 protease, App, HrpA, and NHBA (Tommassen and Arenas, 2017). IgA1 protease and App (Adhesion and penetration protein) both belong to the family of chemotrypsin-like serine proteases and possess conserved positively charged α-domains. These α-domains bind eDNA, contributing to biofilm formation. App is highly conserved in meningococci and is expressed by all *Neisseria* spp. (Hadi et al., 2001). HrpA (haemagglutin/haemolysisn related protein A) is a large exoprotein secreted from Nme *via* a two-partner secretion system involving the HrpB protein (Schmitt et al., 2007). HrpA contains a highly conserved TPS domain and a variable functional domain (Schmitt et al., 2007). HrpA has been shown to play a key role in biofilm formation on human bronchial epithelial cells (Neil and Apicella, 2009). NHBA (*Neisseria* heparin binding antigen) is a surface-exposed lipoprotein ubiquitously expressed by Nme, which can also bind DNA (Arenas et al., 2013a).

### 3.3 Intimate Adherence and Endocytosis

Intimate association of Nme with the epithelial cell results in extensive remodelling of the host cell, creating a meshwork of filipodia-like cellular protrusions in which Nme replicates (Dumenil, 2011). By subverting the microtubule dependent pathway which controls the morphology and function of epithelial cells, Nme enables transcytosis through the host cell without disrupting the tight junctions between cells (Sutherland et al., 2010; Lécuyer et al., 2012). The recruitment of ezrin and the activation of Src tyrosine kinases and cortactin results in restructuring of the host plasma membrane into a cortical plaque enriched in transmembrane proteins such as CD44, ICAM1, VCAM1, epidermal growth factor receptor, the molecular-linker proteins ezrin and moesin, and characterized by the localized polymerization of cortical actin (Carbonnelle et al., 2009; Barrile et al., 2015). Some studies suggest that Nme localises within intracellular vacuoles, adopting a facultative intracellular lifestyle which would normally result in replication and release onto the polar surfaces of the epithelium for further dispersal (Barrile et al., 2015). In support of this pathway, Barrile et al. (2015) observed that Nme usurps the small GTPases such as Rab22a and Rab3, which control the endocytosis and exocytosis pathways usually associated with the polarised transport of transferrin. Eventually, the asymmetrical distribution of the host cell receptors is dysregulated to such an extent that cell polarity is lost, and Nme exits across the basolateral surface of the epithelial cell into the sub-epithelial tissues, where it can cross into the capillaries for systemic disease (Barrile et al., 2015).

The process of intimate adhesion is governed by the interaction of the Opa and Opc invasins and an array of minor adhesins to their cognate receptors on epithelial cells. Intimate adhesion does not occur until the expression of Tfp, and the polysaccharide capsule is downregulated (Virji, 2009) by the CrgA transcriptional regulator, which enables the switch from Tfp-dependent attachment to Tfp-independent intimate adhesion (Deghmane et al., 2000; Deghmane et al., 2002). Induced in a CREN-dependent manner upon cell contact, CrgA negatively regulates the expression of *pilC1*, *pilE* and capsule biosynthesis genes *cssABC* (Deghmane et al., 2000; Deghmane et al., 2002).

#### 3.3.1 The Major Invasin: Opacity Proteins

Opa proteins are structurally variable and highly diverse, with different variants exhibiting tropism for different cell types (Sadarangani et al., 2011). These proteins consist of eight transmembrane β-barrel domains with four surface-exposed loops, of which two are hypervariable and one semi-variable (Sadarangani et al., 2011). These adhesins are encoded by four loci, *opaA*, *opaB*, *opaD*, and *opaJ*, which are subject to independent phase-variation and homologous recombination, contributing to meningococcal antigenic variation (Aho et al., 1991). Opa alleles have been regularly observed at the same locus during global spread spanning decades, indicating that particular meningococcal genotypes encode distinct Opa repertories (Callaghan et al., 2006).

The majority of Opa alleles bind the carcinoembryonic antigen-related cell adhesion molecules (CEACAM) expressed on the surface of a variety of host cell types. Of the host CEACAM repertoire, Nme Opa bind CEACAM1, CEACAM3, CEACAM5 and CEACAM6 (Sadarangani et al., 2011). The binding specificity is governed by ligand interactions between the conserved CEACAM N-domain and two hypervariable loops on the Opa adhesin (Martin et al., 2016). CEACAM1, CEACAM3 and CEACAM6 are expressed on the apical surface of epithelial cells and, due to their GPI-anchor, are directed to cholesterol- and sphingolipid-enriched membrane microdomains (lipid rafts) (Schmitter et al., 2007). Meningococcal binding of CEACAMs initiates membrane microdomain-mediated uptake, which avoids maturation into acidic lysosomes, thus potentiating the development of vacuoles that sustain Nme in the host cell and eventual apical-to-basolateral transport in polarized epithelia (Schmitter et al., 2007). CEACAMs can modulate integrin-mediated cell adhesion at the basolateral surface of the host cell and control exfoliation of host cells from the basement membrane which is a protective mechanism to remove infected host cells. Although it has been shown that Opa-dependent CEACAM engagement prevents exfoliation from the basement membrane in gonococcal models of infection (Tchoupa et al., 2014), this has not been confirmed for Nme.

Some Opa alleles interact with cell-surface associated HSPGs (heparin sulfate proteoglycans), which belong to either the GPI (glycosylphosphatidylinositol)-linked or the transmembrane syndecan family (Hill et al., 2010). HSPG binding regulates many cell functions in a context-dependent manner, but in epithelial cells, it triggers endocytosis *via* multiple pathways which are currently undefined (Sarrazin et al., 2011). Opc which is a 10-stranded β-barrel with five surface exposed loops can also initiate invasion *via* binding to HSPG (Olyhoek et al., 1991). The expression of Opc is controlled at the transcriptional level by phase-variation of a polycytidine tract in the promoter region but the locus is missing from certain lineages including cc11 (Schubert-Unkmeir, 2017). However, the Opa proteins are the predominant invasin at the epithelial surface while Opc has a more dominant role during systemic disease and engagement with endothelial cells (Schubert-Unkmeir, 2017).

#### 3.3.2 Minor Adhesins

The minor adhesins NadA, NhhA, App, MspA, HrpA and NHBA are also involved in nasopharyngeal colonisation and invasion. Although the roles of these minor adhesins are not fully understood, it appears that they re-enforce signalling *via* the endocytic pathway for bacterial uptake into the host cell. NadA binds the epithelial cell receptor β1 integrin, which has an important role in the initiation of endocytosis (Nägele et al., 2011). The *nadA* gene is lineage-restricted, being present in only 5.1% of carriage isolates but present in almost all isolates from cc11, cc8, and cc32 (Comanducci et al., 2004). The expression of NadA is regulated by the *nadR* (aka *farR*) repressor, integration host factor (IHF), the ferric uptake regulatory protein Fur, and a phase-variable tract in the promoter (Metruccio et al., 2009; Cloward and Shafer, 2013). The *nadR* gene is itself regulated by the MtrR repressor (Cloward and Shafer, 2013). NhhA (*Neisseria* hia/hsf homologue A) was shown by Scarselli et al. (2006) to promote adherence of a recombinant NhhA-expressing *E. coli* strain to the epithelium by binding to laminin and heparan sulfate and subsequent binding of these molecules to their epithelial receptors. Additionally, it was shown that adhesion of a MC58 null mutant to epithelial cells was significantly reduced compared to wild-type meningococci (Scarselli et al., 2006). MspA is a third chemotrypsin-like protease which is present in only a subset of lineages (Oldfield et al., 2013). Unlike the related IgA1 and App proteases, MspA has no role in biofilm formation, but like App, it has been shown to bind to epithelial cells (Serruto et al., 2003; Turner et al., 2006) and the mannan and transferrin receptors of dendritic cells (DCs) (Khairalla et al., 2015). NHBA and HrpA, two proteins involved in bacterial aggregation, have also been shown to have functions in mediating attachment to epithelial cells *via* HSPGs (Schmitt et al., 2007; Vacca et al., 2016).

## 4 Systemic Disease

**Figure 3:**
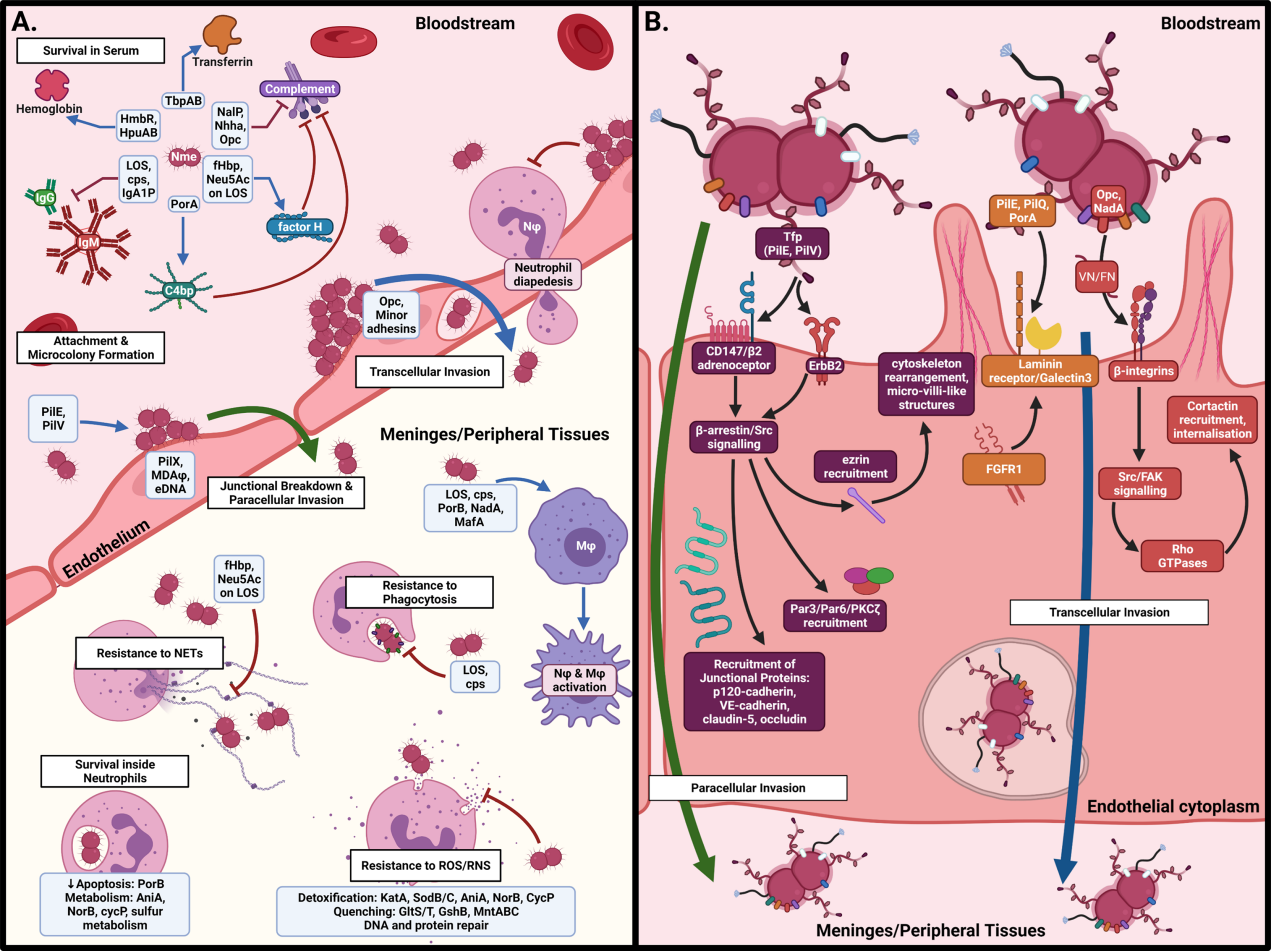
Interactions of *N. meningitidis* in the systemic circulation. **(A)** In the bloodstream, Nme must resist antibody- and complement-mediated killing, acquire iron, and attach to the capillary endothelial surface to form microcolonies. Once attached, Nme possesses several mechanisms to resist the actions of phagocytic cells, including neutrophils and macrophages. **(B)** Binding of endothelial cells occurs by interaction of meningococcal surface structures with their cognate receptors, resulting in cortical plaque formation, transcytosis, and breakdown of tight junctions. This figure was created using Biorender.com.

Once Nme crosses the nasopharyngeal barrier, it encounters a radically different environment to the nasopharynx. In the bloodstream, Nme must contend with different sources of iron and other metabolites, antibody- and complement-mediated killing, circulating immune cells, and the shear stress produced by blood flow. To cause meningitis or septicaemia, meningococci must attach to endothelial cells in the blood-brain barrier (BBB) and peripheral vasculature, respectively. Once attached, Nme resists the influx of phagocytic cells to infected sites, modulates the local thrombotic response, and cause the blood vessels to become leaky, allowing dissemination into the meninges or surrounding tissues, thus leading to the syndromes of meningitis and purpura fulminans, respectively. The interactions of Nme with the host once inside the systemic circulation are detailed in **Figure 3** and in the following sections.

### 4.1 Survival in the Bloodstream

The complement system is the critical host defence against meningococci once they cross the epithelium, as evidenced by the fact that complement-deficient individuals are at a highly increased risk for IMD, and that an intact complement system is required for the killing of Nme by whole blood (Lewis and Ram, 2020). In addition, activation by the complement pathway is essential to an efficient anti-bacterial response by host neutrophils (Krüger et al., 2018). The mammalian complement system is activated *via* three pathways, all of which converge on the production of a C3 convertase which cleaves complement proteins C3, C4, and C5 into their active forms and leads to the downstream formation of the membrane attack complex (MAC), which disrupts bacterial cells membranes. The three pathways are termed the classical pathway (CP), which proceeds by binding of specific IgG and IgM antibodies to bacterial targets; the lectin pathway (LP), which proceeds from the binding of mannose binding lectin (MBL) to surface carbohydrates; and the alternative pathway (AP), which results from spontaneous ‘tickover’ of C3 into C3(H_2_O) which is subsequently converted into the C3 convertase C3(H_2_O)Bb by factor B and factor D (Lewis and Ram, 2020). The primary targets of complement deposition on the meningococcal surface are the LOS, porins, and Opa proteins (Ram et al., 2003; Lewis et al., 2008). MBL is also capable of direct binding to PorB and Opa proteins in order to activate the AP (Estabrook et al., 2004).

The expression of capsule is required for resistance to complement, and meningococci lacking a capsule are rarely recovered from disease settings in immunocompetent patients. In particular, expression of one of the four sialic acid containing capsules (serogroups B, C, W, or Y) has been shown to reduce the deposition of C4b and activation of the CP by blocking the binding of IgG and IgM antibodies to multiple surface-expressed proteins (Agarwal et al., 2014). Expression of sialic acid on erythrocytes is a known mechanism to block complement deposition on host cells, and the expression of sialic acid in capsule presumably functions in a similar fashion (Langford-Smith et al., 2015). Different capsular polysaccharides modulate complement in different ways, with expression of capsule from B, C, W, and Y reducing CP activation, serogroup A capsule having no effect on CP or AP activation, and serogroup W and Y capsules somewhat paradoxically increasing AP activation by deposition of C3b onto the capsule itself (Ram et al., 2011). O-acetylation, which occurs in multiple capsule types and is phase-variable in some serogroups, has also been shown to modify serum bactericidal activity, having a protective effect in serogroup C isolates but sharply enhancing the immunogenicity of the serogroup A capsule (Tuomanen et al., 2001; Berry et al., 2002). Meningococcal strains over-expressing capsule display increased serum resistance, and variation of capsule expression may represent a mechanism of immune evasion in Nme. Capsule expression is upregulated by temperature via a thermosensor secondary structure in the 5’UTR of the mRNA of *cssABC* operon (Loh et al, 2013). Variation in the repeats comprising the stem-loop of the thermosensor and insertion of the IS1301 element in this location modulate expression of sialic acid synthesis affecting capsule polysaccharide and LOS sialylation (Uria et al., 2008; reviewed in Tzeng et al., 2016).

Although capsule is essential for resistance to human serum, multiple studies have demonstrated that variations in LOS structure are also responsible for modulating resistance to complement. Meningococcal LOS can be 12 structures termed immunotypes, based on the presence and phase-variation states of the glycosyltransferase genes involved in the synthesis of the α-chain and the genes involved in the decoration of the LOS inner core (Bartley and Kahler, 2014; Mubaiwa et al., 2017). The LOS α-chain is partially decorated with sialic acid (Neu5Ac) by the Lst sialyltransferase, which uses CMP-Neu5Ac scavenged from the host serum or endogenously synthesised by stains expressing sialic acid containing capsules (i.e. serogroups B, C, W, and Y). The two LOS α-chain structures which may be sialylated in Nme are LNnT [Gal(β1-4)GlcNAc(β1-3)Gal(β1-4)Glc] *via* an α2-3 linkage, and the P*^K^*-like antigen (the L1 immunotype) *via* an α2-6 linkage (Wakarchuk et al., 1998; Gulati et al., 2005). A sialylated LNnT α-chain has been shown to enhance resistance of encapsulated strains to human serum (Kahler et al., 1998). Although Lst expression in *N. gonorrhoeae* is regulated by CrgA, the insertion of a Correia element in the promoter region of meningococcal *lst* has resulted in an alternate promoter not subject to CrgA-based regulation (Matthias and Rest, 2014). Sialylation of LOS is controlled in part by the availability of CMP-Neu5Ac, and therefore is subject to similar regulatory mechanisms as capsule expression in serogroup B, C, W and Y strains (see above). Co-regulation of sialic acid synthesis and expression of *lst* occurs by temperature shift as the 5’UTR of the *lst* mRNA contains a thermosensitive riboswitch (Loh et al., 2013). In addition to α-chain structure and sialylation, decoration of the LOS inner core also modulates the complement response. Substitution of the Heptose II residue of the LOS inner core (HepII) with O-6 linked PEA, but not O-3 linked PEA, carried out by the PEA-transferases Lpt6 and Lpt3 respectively, is associated with increased deposition of C4b on LOS when an LNnT α-chain is present (Ram et al., 2003). HepII substituted with O-3 linked PEA may also undergo C4b deposition when the α-chain is truncated (Ram et al., 2003). Since both *lgtG* and *lpt6* are found on genomic islands, strain variation in LOS inner core structure may contribute to the difference in pathogenicity of different meningococcal lineages (Mackinnon et al., 2002; Kahler et al., 2005). Phase-variation of LgtG, which preferentially adds a Glc residue in place of the O-3 linked PEA added by Lpt3, might also contribute to varying serum sensitivity in Nme (Berrington et al., 2002; Kahler et al., 2005).

In addition to surface carbohydrate expression, Nme possesses a number of surface proteins that are able to modulate complement deposition and contribute to serum resistance (reviewed in Lewis and Ram, 2020). The NalP protease is able to cleave human C3 in both its membrane-bound and secreted forms, resulting in the degradation of the generated C3 fragment by host factors and reduced C3 deposition on the meningococcal surface (Del Tordello et al., 2014). The host complement inhibitor C4 binding protein (C4bp) is recruited by meningococcal PorA, resulting in the inactivation of C4b and irreversible dissociation of the C4b2a convertase and inhibition of the CP. PorA expressing strains are more resistant to serum, however, C4bp recruitment is inhibited by capsule expression (Jarva et al., 2005). NHBA has been shown to increase serum resistance *via* binding to host heparin (Serruto et al., 2010). Both NalP and lactoferrin are capable of cleaving NHBA, and both the membrane-bound and secreted forms have similar heparin-binding activity (Serruto et al., 2010). Another component of the extracellular matrix, vitronectin, has been shown to inhibit complement activation and is bound by multiple meningococcal antigens, including NhhA and Opc, to reduce the formation of the MAC on meningococcal cells and increase serum resistance (Sa et al., 2010; Griffiths et al., 2011).

A particularly important feature of meningococcal complement resistance is the ability to bind human factor H (fH), which acts as a cofactor in the cleavage of C3b into its inactive form by factor I and carries out irreversible inactivation of the C3bBb C3 convertase, thereby playing a large role in the inhibition of the AP (Schneider et al., 2006). In gonococci, direct binding of fH to α2-3 sialylated LNnT is observed in strains expressing gonococcal PorB, however, meningococcal PorB cannot stabilise this interaction, and thus direct binding of fH is not observed (Madico et al., 2007; Lewis and Ram, 2020). Instead, fH may bind C3b fragments deposited on sialylated meningococcal LOS in a manner similar to the binding of glycosaminoglycans and C3 fragments on host cells (Lewis et al., 2012). Despite a low binding affinity, PorB binds fH at a rate that is clinically relevant (Lewis et al., 2013; Giuntini et al., 2015). Neisserial surface protein A (NspA) is also capable of binding fH in a manner influenced by LOS structure: a truncated α-chain or sialylation of LOS is associated with increased fH binding by NspA (Martin et al., 1997; Vandeputte-Rutten et al., 2003; Lewis et al., 2010). Finally, Nme expresses a fH binding protein (fHbp), responsible for the recruitment of fH to the meningococcal surface to inhibit AP activation (reviewed in Principato et al., 2020). Significant structural variation of fHbp exists among Nme isolates, with three major families (variant 1, variant 2, and variant 3) being described. Hundreds of sub-variants within each family exist, many of which are associated with ST (Masignani et al., 2003; Bambini et al., 2009; Brehony et al., 2009). fHbp is expressed by two independent promoters, one bicistronic upstream of the proximal gene to *fhbp*, and a dedicated monocistronic *fhbp* promoter which is under the control of the fumarate and nitrate reductase regulon and responds to anaerobic conditions/decreased oxygen concentrations (Oriente et al., 2010). Expression of *fhbp* has also been shown to increase under iron-replete conditions in most strains (Sanders et al., 2012). Expression of fHbp can vary up to 15-fold between strains based on the genetic sequence of the promoter region and is correlated with serum bactericidal activity (Biagini et al., 2016). SNPs in the signal peptide sequence of *fhbp* have also been shown to modulate trafficking of the mature protein to the membrane, altering the levels of surface-available fHbp and resistance to antibody-mediated killing (da Silva et al., 2019).

Lastly, glycosylation of surface-exposed proteins, especially the PilE subunit of Tfp, is another means of avoiding adaptive immunity by masking the surface of the bacteria from opsonisation. Meningococcal Tfp exists as one of two major classes, class I and class II, the former of which undergoes rapid antigenic variation by recombination of *pilE* with repeating cassettes of *pilS* pseudogenes, and the latter of which is invariant (Aho et al., 1997; Wörmann et al., 2014). PilE in class I expressing strains possess a single glycosylation site, whereas class II pilin display 2-5 glycosylation sites depending on the proteoform (Gault et al., 2015). The additional glycosylation sites on class II pili may provide an alternate form of immune evasion given the invariant nature of PilE in these strains (Gault et al., 2015). The specific glycans added to PilE are determined by the presence/absence of genes encoding for the synthesis and transfer of glycan residues onto the glycan chain extending from the UDP lipid carrier on the inner membrane (Bartley and Kahler, 2014). Synthesis of the initial sugar in the glycan chain is carried out by PglC, PglD, and either PglB or PglB2, with the allele of *pglB* determining whether the sugar added is diNAcBac or GATDH, respectively (Bartley and Kahler, 2014). Subsequent extension of the glycan chain into di- or tri-saccharides is carried out by PglA and PglE, resulting in a di-galactose addition, or by PglH/H2, which results in the addition of either Glc or GlcNAc, respectively (Power et al., 2003; Børud et al., 2014). Both di- and tri-saccharide glycans may be mono- or di-acetylated by the PglI O-acetyltransferase (Anonsen et al., 2017). The mature glycan is transferred onto PilE or other proteins by the PglO (aka PglL) oligosaccharyltransferase (Musumeci et al., 2013). Microheterogeneity of the proteoglycome is generated through phase-variation and polymorphisms in the *pgl* locus of Nme (Børud et al., 2018). Such heterogeneity is proposed to play an important role in immune evasion, and variation in the pilin glycans expressed by meningococcal strains has been demonstrated to differ both before and after accidental human passage and between strains carried by the same individual within a short time period (Omer et al., 2011; Børud et al., 2018).

In addition to bacterial mimicry of host structures and recruitment of host immunoregulatory proteins, meningococcal metabolism also plays an important role in impeding complement deposition and activation during systemic infection with Nme. Lactate uptake by the lactate permease LctP has been shown to be critical for resistance to complement as lactate is an entry metabolite into the sialic acid biosynthesis pathway required for capsule and LOS sialylation (Exley et al., 2005a; Exley et al., 2005b). Sulfur metabolism also plays multiple roles in virulence. A mutant lacking cysteine binding protein was internalised by endothelial cells at a rate 100-fold lower than that of wild-type, and depletion of cysteine and other sulfur sources triggers increased membrane blebbing (Gerritzen et al., 2018; Takahashi et al., 2018). Nme shed the outer membrane as blebs which play multiple roles in virulence. Shedding the outer membrane rapidly and irreversibly removes bound complement from the bacterial surface, preventing MAC insertion and lysis. Shed blebs are also known to fuse with the membranes of surrounding host cells, delivering cytotoxins which further induce the inflammatory response and misdirect phagocytic cells to locations distant from the microcolony (Kaparakis-Liaskos and Ferrero, 2015).

### 4.2 Endothelial Colonisation

In the bloodstream, meningococcal microcolony formation and adherence phenotypes are opposed by the high-pressure environment and shear stress exerted by blood flow. Mairey et al. (2006) demonstrated using a laminar-flow model that the only vessels in which shear stress levels are low enough to allow microcolony formation are capillaries and small conducting vessels. At these sites, the transient and heterogeneous nature of perfusion allows meningococci to undergo initial attachment to endothelial cells and to form microcolonies in a manner similar to colonisation at the nasopharyngeal epithelium (see above). This evidence is supported by post-mortem histology performed on an untreated meningitis case in which microcolonies of Nme were observed in the cerebral capillaries (Pron et al., 1997). Similarly, the colonisation of peripheral capillaries by Nme has been shown to occur in skin lesions of patients with purpura fulminans and in human skin-graft models in mice (Sotto et al., 1976; Harrison et al., 2002; Join-Lambert et al., 2013).

#### 4.2.1 Paracellular Transport

In contrast to interactions at the epithelial surface, where the receptor for Tfp is still unknown, the interactions between the pilus and human brain endothelial cells are well established (Lécuyer et al., 2012). The meningococcal major pilin, PilE, and minor pilin, PilV, bind the CD147 receptor *via* recognition of a triantennary sialylated poly-N-acetyllactosamine–containing N-glycan (Bernard et al., 2014; Le Guennec et al., 2020). CD147 is complexed to the β_2_-adrenoceptor (β_2_AR) on endothelial surfaces, and Tfp binding induces biased activation of the β_2_AR and subsequent activation of β_2_-arrestins, stimulating rapid recruitment of cytoskeleton-associated and signalling proteins to remodel the plasma membrane underneath the newly forming microcolony (Coureuil et al., 2009; Mikaty et al., 2009). In particular, recruitment of ezrin and moesin to the site of adhesion results in actin polymerisation and the formation of microvilli-like structures, and recruitment of α-Actinin4 drives increases in the local density of CD147-β_2_AR complexes in order to increase the strength of microcolony adhesion (Maissa et al., 2017). The recruitment of β_2_AR signalling partners such as Src tyrosine kinases; p120-catenin and VE-cadherin (adherens junctional proteins); zonula occludens-1, claudin-5, and occludin (tight junctional proteins); and the Par3/Par6/PKCζ polarity complex to the site of bacterial attachment to form a cortical plaque results in weakening and eventual failure of the tight-junctions between endothelial cells, allowing the passage of Nme paracellularly into the meninges in the case of meningitis, or the peripheral tissues in the case of meningococcaemia (Maissa et al., 2017). In addition to CD147, Tfp bind laminin receptor precursor 1 (LAMR1/37LRP) co-localised with galectin-3 on the surface of hBMVECs *via* the major pilin, PilE, and the PilQ secretin (Alqahtani et al., 2014). The mature Laminin receptor (67LR) is recognized by PilQ and PorA and is a common receptor shared by *S. pneumoniae* and *H. influenzae* (Orihuela et al., 2009). Nme has also been shown to recruit both forms of fibroblast growth factor receptor 1 (FGFR1), which co-localise with 37LRP, and to a lesser extent, 67LR. Knockdown of FGFR1 using siRNA resulted in a significant reduction in the adherence and invasion of Nme into endothelial cells, suggesting an important role for this protein in meningococcal virulence (Azimi et al., 2020).

#### 4.2.2 Transcellular Transport

Once attached to the endothelial surface, microcolony formation proceeds in a similar manner to epithelial binding (see above), and meningococcal aggregates begin to occlude the vessels they occupy (Manriquez et al., 2021). In addition to the paracellular route of invasion resulting from tight junction breakdown initiated by Tfp, meningococci can cross the endothelium *via* transcytosis. The most important mediator of this process at the endothelial surface is Opc, in contrast to the shared role played by Opc and the Opa proteins at the epithelial surface (Virji et al., 1993a). Opc binds the endothelial surface *via* binding to vitronectin and fibronectin, following which binding of αvβ3-integrin or α_5_β_1_-integrin (respectively) occurs (Unkmeir et al., 2002b). Vitronectin is the preferred substrate for Opc binding. Bacterial uptake by endothelial cells *via* the integrin pathway follows and is mediated by an interplay between Src, focal adhesion kinase, and cortactin (Slanina et al., 2010; Slanina et al., 2012). The binding of vitronectin occurs *via* a heparin bridge (De Vries et al., 1998; Tuomanen et al., 1999) or directly *via* binding to the sulfated tyrosines on these proteins (Sa et al., 2010). Tfp-based binding of meningococci induces transient increases in cytosolic Ca^2+^ in endothelial cells, resulting in the translocation of acid sphingomyelinase (ASM) to the surface of the cell and the development of ceramide-rich lipid micro-domains at attachment sites (Simonis et al., 2014; Peters et al., 2019). Opc-mediated internalisation of Nme has been shown to be directly dependent on the levels of ASM and ceramide in these micro-domains, and the ability of Nme to induce micro-domain formation is higher in more invasive strains (Simonis et al., 2014).

The minor adhesins have also been demonstrated to play a role in the transcytosis of meningococci across endothelial barriers. The App and NadA autotransporters have both been shown to increase adhesion to hBMVECs, and a recent study demonstrated that meningococci treated with anti-NadA antibodies exhibit reduced transcytosis across a model of the BBB (Turner et al., 2006; Serruto et al., 2010; Kulkarni et al., 2020). Meningococcal IgA1 protease has been shown to cleave LAMP-1, a major integral glycoprotein of human lysosomes. During attachment, Tfp and Opc-induced CA^2+^ transients trigger exocytosis of lysosomes, bringing LAMP-1 to the surface where it may be cleaved by IgA1 protease (Ayala et al., 2001). A major outer membrane protein, P.IB, has been shown to interact with endothelial cells, but the mechanism is as yet unknown (Kánová et al., 2018). Recently, a role for dynamin and clathrin-mediated endocytosis in the uptake of Nme by endothelial cells has been observed (Herold et al., 2021). Interestingly, the process was only dependent on dynamin in the absence of the meningococcal capsule, whereas Arp2/3 actin polymerisation was shown to be more important for the uptake of wild-type cells.

## 5 Inflammatory Potential and Immune Resistance

### 5.1 Immune Stimulation by the Meningococcus

In late-stage IMD cases, systemic infection by the meningococcus causes rapid and exacerbated activation of the host’s innate immune response, producing unregulated systemic inflammation, dysregulation of coagulation, and severe widespread vascular injury (Pathan et al., 2003). This systemic inflammatory cascade is ultimately what leads to the progression of fulminant sepsis and meningitis in IMD patients, and eventually multi-organ failure and death. Recognition of Nme by multiple human cell types is mediated by pattern recognition receptors (PRRs), which recognise pathogen-associated molecular patterns (PAMPs) common to multiple species of pathogen.

The most important and well-studied PRRs on human cells which recognise Nme are the toll-like receptors (TLRs) (reviewed in Johswich, 2017). Meningococcal LOS is a classical activator of the inflammatory response and is recognised by TLR4 (Pridmore et al., 2001; Zughaier et al., 2004). The affinity of the lipid A to the TLR4 receptor and hence the stimulation of the cytokine response is dependent upon the decoration of the lipid A headgroups with PEA and the distribution and length of the fatty acyl chains (Kahler et al., 2018). Examination of various strain collections indicates that there is considerable micro-heterogeneity of the lipid A pyrophosphorylation which corresponds to the inflammation potential of lipid A (John et al., 2020). TLR4 is also capable of recognising several meningococcal surface proteins, including NhhA and PBP2 (Plüddemann et al., 2009; Hill et al., 2011; Sjölinder et al., 2012). TLR2 in complex with TLR1 recognises meningococcal capsule and surface proteins including PorB, NhhA, and fHbp (Massari et al., 2003; Zughaier et al., 2004; Luo et al., 2016; Wang et al., 2016). TLR9 is located within endosomes and recognises CpG DNA, which is common in bacteria but not in mammalian cells (Mogensen et al., 2006; Magnusson et al., 2007). The intracellular Nod-like receptors (NLR) recognise fragments of peptidoglycan liberated from the meningococcal cell wall upon phagocytosis of Nme (Girardin et al., 2003a; Girardin et al., 2003b). Recognition of additional Nme surface structures by host cells is mediated by a variety of other receptors. Binding of carbohydrate structures is mediated by receptors including MBL, the mannose receptor DC-SIGN, surfactant proteins, siglecs, ficolins, and galectins, whereas meningococcal proteins and peptide fragments may be recognised by the N-formyl peptide receptor and scavenger receptors (Johswich, 2017). Upon the binding of PRRs to their corresponding PAMPs, activation of intracellular signalling pathways (primarily *via* NF-κB signalling) results in the upregulation of genes for the expression of cytokines and chemokines, maturation of immune cells such as DCs, initiation of phagocytosis, and modulation of cell death *via* apoptotic pathways depending on the cell type.

The ultimate result of PRR activation by meningococcal PAMPs varies during different stages of meningococcal disease. During colonisation, a controlled local inflammatory response is elicited by the interaction of Nme with both epithelial cells and resident DCs, resulting in the production of neutrophil chemoattractants including IL-8, C5a and hepoxilin A-3, which initiate firm adhesion of circulating neutrophils and infiltration of the epithelium in order to clear the infection (Stephens et al., 1983; Johswich et al., 2013; Filippi, 2019). The inflammatory response produced by the body in response to systemic infection during IMD is, by contrast, enormous (Johswich, 2017). High levels of pro-inflammatory cytokines (including IL-1α, IL-1β, IL-2, IL-6, MIF), chemokines (including IL-8, MCP-1, MIP-1α, MIP-1β), factors stimulating neutrophil and monocyte activation and maturation (including G-CSF, GM-CSF, IFN-γ, TNF-α), and complement components and activation products (including C1q, MBL, C3a, iC3b, C5a, sC5b-9, CFH) are detectable in both the CSF and serum of patients during meningococcal sepsis and meningitis (Mook-Kanamori et al., 2014; Johswich, 2017).

LOS is a key activator of inflammation, causing high levels of cytokine release in multiple cell types including DCs, macrophages, and meningeal cells (Clements et al., 2001; Christodoulides et al., 2002; Zughaier et al., 2004). Several meningococcal surface proteins, including PorB, the autotransporter NadA, and the MafA component of the MafAB toxin-antitoxin system have also been shown to directly stimulate the production of cytokines in human cell lines (Singleton et al., 2005; Massari et al., 2006; Mazzon et al., 2007; Franzoso et al., 2008; Káňová et al., 2019). In addition to cytokine release, meningococcal proteins may modulate apoptosis in host immune cells. The autotransporters MspA and App have both been shown to be internalised by DCs, following which they are trafficked to the nucleus causing a dose-dependent increase in caspase mediated apoptosis (Khairalla et al., 2015). In contrast, meningococcal PorB has been demonstrated to insert itself in the mitochondrial membrane of host cells, altering mitochondrial depolarisation and protecting cells from apoptosis (Massari et al., 2003; Peak et al., 2016). NhhA has been shown to have multiple anti-inflammatory effects. When NhhA is used to stimulate monocyte maturation, a profile of cytokines geared towards an anti-inflammatory and pro Th2 response (including IL-10, CCL17, CCL18, CCL22) are released (Wang et al., 2016). NhhA has also been shown to increase the rate of macrophage apoptosis (Sjölinder et al., 2012).

### 5.2 Professional Phagocytes and Resistance to Phagocytosis

A key consequence of the inflammatory response triggered by infection with Nme is the maturation and recruitment of immune cells. The majority of mononuclear phagocytes resident in the human nasopharynx are plasmacytoid and myeloid DCs, with a smaller population of resident monocytes/macrophages (Vangeti et al., 2018). During the colonisation of the nasopharynx with Nme, bacteria that successfully cross the nasopharyngeal epithelium engage basolateral Toll-like receptors (TLRs), activating NF-κB signalling and the release of chemoattractant chemokines including IL-8 (Filippi, 2019). Assembly of the membrane attack complex (MAC) on bacterial membranes results in the conversion of C5 and the release of C5a, which also has chemoattractant properties (Filippi, 2019). An increasing chemoattractant gradient stimulates the activation of circulating neutrophils, increasing expression of CD11b and CD18, leading to firm adhesion to local endothelial cells, formation of endothelial docking structures by reorganisation of ICAM-1 and JAM-A on the endothelial surface, and neutrophil diapedesis into local tissues by either the paracellular or transcellular route (van Buul et al., 2007). Once transmigration has occurred, neutrophils and tissue resident DCs and monocytes are activated by contact with Nme through multiple pathways and initiate bacterial killing by phagocytosis, production of ROS, nitric oxide, CAMPs, and in the case of neutrophils, the production of neutrophil extracellular traps (NETs) (Urban et al., 2006; Filippi, 2019).

#### 5.2.1 Dendritic Cells and Macrophages (Monocyte Derived Cells)

DCs activate upon contact with Nme, stimulating the release of the proinflammatory cytokines IL1-β, IL-6, IL-8, TNF-α, IFN-γ, and GM-CSF (Kurzai et al., 2005). Neisserial LOS has been identified as a major mediator of the DC proinflammatory response. The expression of LOS containing sialylated LNnT reduce the adherence and subsequent phagocytosis of Nme (Clements et al., 2001; Unkmeir et al., 2002b; Kurzai et al., 2005). Capsule expression has also been shown to inhibit phagocytosis in DCs. Interestingly, capsule expression and variation in LOS structure have not been shown to alter the release of pro-inflammatory cytokines, although capsule expression has been shown to reduce the level of the regulatory cytokine IL-10 (Unkmeir et al., 2002b; Kurzai et al., 2005). Multiple meningococcal surface proteins have been shown to play a role in modulating the response of DCs to infection. The porins PorA and PorB have been shown to induce the maturation of monocyte-derived DCs, inducing chemokine release (IL-8, RANTES, MIP-1α, MIP-1β) and the expression of DC markers (CD40, CD54, CD80, CD86, MHC-II) (Singleton et al., 2005; Khairalla et al., 2015). PorA also increased the capacity of DCs to activate both naïve and memory T-cells but inhibited the production of IL-12p70, thereby directing activated T-cells towards a Th2 response (Al-Bader et al., 2004). The response to PorB was shown to be dependent on recognition by TLR2/1 and subsequent activation of MyD88 signalling (Singleton et al., 2005; Massari et al., 2006). The minor adhesins App and MspA have both been shown to bind mannose receptor and transferrin receptor on DCs, traffic to the nucleus, and induce a dose-dependent increase in DC death *via* caspase-dependent apoptosis (Khairalla et al., 2015). NadA, which is expressed predominantly by hyperinvasive lineage cc11 isolates, has also been shown to interact with DCs. Stimulation of DCs with NadA strongly upregulated DC maturation markers (CD83, CD86, CD80, HLA-DR) and resulted in moderate cytokine secretion (Mazzon et al., 2007).

Tissue resident macrophages represent a critical component of the innate immune response thanks to their roles in antigen presentation and the initial cellular antibacterial response (Escobar et al., 2018). Macrophages are activated by recognition of PRRs, including the TLRs and scavenger receptors such as scavenger receptor-AI/II (SR-A) and macrophage receptor with collagenous domain (MARCO). Activation of macrophages by Nme occurs *via* binding of the KDO residues of meningococcal LOS to TLR4, binding of PorB to TLR2, and binding of multiple surface-exposed proteins to SR-A and MARCO (Johswich, 2017). Opsonisation of Nme by MBL, a key activator of the LP of complement, has also been shown to accelerate the uptake of Nme into macrophages (Jack et al., 1998; Jack et al., 2005). Nme has several adaptations to resist killing by macrophages. As with most cell types, expression of the capsule has been shown to reduce phagocytosis and inhibit the initial fusion of the phagosome with the lysosome (Read et al., 1996). Resistance to the production of NO by macrophages is mediated by the nitric oxide reductase NorB and, to a lesser extent cytochrome c’ (CycP) (Stevanin et al., 2005). Detoxification of NO by NorB has also been shown to downregulate the production of pro-inflammatory cytokines by macrophages, likely contributing to survival in these cells (Stevanin et al., 2007). Multiple surface proteins of Nme have been found to downregulate the apoptosis of macrophages, including NadA, NhhA, NorB, CycP, and PorB, the latter of which inhibits apoptosis in multiple cell types by inserting into the mitochondrial membrane, preventing mitochondrial depolarisation and activation of caspase-9 and -3 dependent apoptosis (Massari et al., 2000; Massari et al., 2003; Tunbridge et al., 2006; Franzoso et al., 2008; Wang et al., 2016). Although Nme prefers oxygen respiration, several studies have indicated that the expression of a denitrification pathway allows the meningococcus to utilise nitric oxide as an energy source and that this ability may aid the survival of Nme intracellularly (Tunbridge et al., 2006; Stevanin et al., 2007).

#### 5.2.2 Recruited Neutrophils

Neutrophils recruited to infected vessels are a key part of the host defence against systemic bacterial infections, and an inflammatory infiltrate consisting primarily of neutrophils and macrophages is diagnostic for a range of bacterial meningitis pathogens, including Nme (Sotto et al., 1976; Harrison et al., 2002; Coureuil et al., 2017; Shahan et al., 2021). Clinical IMD cases are marked by early signs of neutrophil activation, including increased CD11b and CD18 expression and shedding of CD62L (L-selectin) (Peters et al., 2003). Neutrophils are recruited to arterioles, capillaries and venules containing attached Nme, however, it was recently demonstrated that neutrophil populations in these sites are heterogenous (Manriquez et al., 2021). Manriquez et al. (2021) showed that while neutrophils were recruited in large numbers to venules in human skin grafts in a mouse model of IMD, the level of adherent neutrophils in arterioles and capillaries was greatly reduced, leading to insufficient clearance of Nme from these vessels. The colonisation of these sites, therefore, represents a mechanism by which Nme may evade killing by neutrophils. Nme may also directly reduce the recruitment of neutrophils to infected vessels to evade killing. While both encapsulated and unencapsulated meningococci induce shedding of L-selectin by neutrophils (leading to increased adhesion at peripheral sites), a meningococcal mutant lacking a long-chain LOS induced greater-neutrophil adhesion than the wild-type strain, suggesting that LOS may play a role in inhibiting neutrophil recruitment (Klein et al., 1996).

Phagocytosis of Nme by neutrophils is primarily triggered by the deposition of complement factors or opsonising antibodies on the bacterial surface and is resisted by capsule, LOS, and surface proteins (described in detail in Section 5.2.1). Non-opsonic phagocytosis can be carried out by direct binding of neisserial surface structures to receptors on neutrophils surfaces. In the gonococcal model, binding of gonococcal Opa proteins to CEACAM3 (but not CEACAM1 or CEACAM6) results in non-opsonic phagocytosis followed by oxidative burst and degranulation (Sarantis and Gray-Owen, 2007; Sanders et al., 2012). Since meningococcal Opa proteins are capable of binding CEACAM3, it is probable that Nme may be uptaken by neutrophils in a similar manner (Sarantis and Gray-Owen, 2007; Sanders et al., 2012). Sialylation of meningococcal LOS has been shown to inhibit non-opsonic phagocytosis in some Nme strains (Estabrook et al., 1998). Neisserial porins play a key role in resistance to neutrophils, inhibiting opsonic phagocytosis, degranulation, and phago-lysosome fusion (Bjerknes et al., 1995). The inhibition of apoptosis by PorB observed in epithelial cells and DCs is likely to occur in neutrophils as well, representing a probable mechanism by which those meningococci that survive within neutrophils may extend their lifespan (Criss and Seifert, 2012; Peak et al., 2016). NETs produced by neutrophils in response to infection are deployed to immobilise and kill bacteria by depriving them of critical nutrients and by CAMP- and ROS-mediated killing. The binding of meningococci to NETs is partially mediated by Tfp (Lappann et al., 2013). Given the high affinity of meningococcal pilin for DNA *via* the ComP subunit (Cehovin et al., 2013), this interaction is likely mediated by ComP. Both Nme and outer membrane blebs are capable of inducing the production of NETs, and the release of blebs results in misdirection/depletion of NETs to protect meningococci (Lappann et al., 2013). EptA-mediated modification of Lipid A headgroups with PEA is also important for the resistance to NETs (Lappann et al., 2013). Such resistance is not due to decreased binding to NETs, but rather resistance to the action of NET-bound cathepsin G. Capsule plays a role in resistance to NETs, as indicated by increased binding of capsule mutants by NETs. Zinc uptake *via* ZnuD is also important for survival in NETs, and NETs may withhold zinc from bacteria bound to them (Lappann et al., 2013).

The production of ROS and reactive nitrogen species (RNS) by both neutrophils and macrophages causes large amounts of damage to bacterial proteins, lipids, and DNA, ultimately resulting in the destruction of phagocytosed pathogens (Kozlov et al., 2003; Imlay, 2013). To resist killing by ROS and RNS, meningococci possess a range of proteins that detoxify ROS, including catalase, cytochrome *c* peroxidase and two superoxide dismutases (reviewed in Criss and Seifert, 2012). In addition to detoxification, quenching of ROS is also used to protect Nme against ROS-mediated damage. Nme has two glutamine uptake systems, GltT and GltS, which work in tandem with the glutathione synthase GshB to acquire L-glutamine and convert it into glutathione in order to further quench ROS (Talà et al., 2011). Manganese has been shown to scavenge the superoxide radical and dismutate H_2_O_2_ in the presence of bicarbonate (Archibald and Fridovich, 1982; Stadtman et al., 1990), and the meningococcal Mn uptake system MntABC has been shown to play a significant role in the resistance of Nme to ROS (Seib et al., 2004). Resistance to the production of RNS by macrophages is mediated by the nitric oxide reductase NorB and, to a lesser extent, cytochrome c’ (CycP) (Stevanin et al., 2005). Detoxification of NO by NorB has also been shown to downregulate the production of pro-inflammatory cytokines by macrophages, likely contributing to survival in these cells (Stevanin et al., 2007).

In addition to detoxification, repairing damaged DNA and proteins is critical to cell viability and survival of Nme in phagocytes. Neisserial exonuclease (NExo) and neisserial apurinic/apyrimidinic endonuclease (NApe) have both been shown to contribute to survival in human neutrophils *via* their ability to remove damaged abasic residues from DNA (Carpenter et al., 2007). DNA repair by these enzymes is backed up by a redundant network of enzymes, including the bifunctional DNA glycosylase/glycolyases Nth and MutM, making the meningococcus robust to DNA damage by ROS (Nagorska et al., 2012). The DinG helicase has also been shown to increase survival under oxidative stress due to its role in double-stranded break repair (Frye et al., 2017). Repair of damaged proteins occurs *via* several pathways. Methionine sulfoxide residues on damaged proteins are repaired by an outer membrane lipoprotein called PilB. PilB comprises two fused methionine sulfoxide reductase domains (MsrA/B domains) fused to an N-terminal thioredoxin (Trx) domain. Electrons required to reduce methionine sulfoxide into methionine are channelled through the Trx domain *via* the inner membrane protein DsbD (Brot et al., 2006). Damage to cysteine residues occurs primarily by the breakage of thiol-disulfide bonds critical for protein structure and function. The Dsb proteins, involved in the oxidation and isomerisation of thiol-disulfide bonds, repair this damage in Nme and ensure correct folding of their target proteins (Piek and Kahler, 2012). Nme contains three DsbA homologues: DsbA1, DsbA2, which among others is involved in the formation of disulphide bonds in the PilE and PilQ subunits of Tfp, and DsbA3, which catalyses disulphide bond formation in the LOS PEA-transferase EptA (Sinha et al., 2004; Tinsley et al., 2004; Sinha et al., 2008; Piek et al., 2014). Each of these DsbA proteins in Nme is re-oxidised by the inner membrane protein DsbB (Piek and Kahler, 2012). The isomerisation pathway consists of DsbD, which transfers electrons to DsbC, allowing DsbC to reshuffle thiol-disulfide bonds in proteins containing multiple cysteine residues (Piek and Kahler, 2012). Interestingly, DsbD has been identified as essential in Nme (Kumar et al., 2011).

## 6 Evolution of Commensalism and Pathogenicity

Nme is a useful species to examine the evolution of virulence as it contains both non-invasive genetic lineages and hyperinvasive lineages which differ in their capacity to cause IMD. The evolution of virulence in a pathogen is a dynamic continuum between the acquisition of patho-adaptive mutations and fitness in any given environmental niche (Diard and Hardt, 2017). The adaption towards virulence by a pathogen may result in an ecological advantage such as improved colonisation and transmission through the human population and thereby provides a competitive advantage against other strains without this feature. In the case of Nme, IMD is considered a dead-end in the transmission cycle and provides no obvious competitive advantage to genetic lineages with this trait. In theory this should ultimately result in the slow extinction of the hyperinvasive lineages over time. However, two evolutionary forces oppose this process: acquisition of traits *via* horizontal gene transfer (HGT) and the evolution of hypervariable and hypermutable loci (De Ste Croix et al., 2020). Dependent upon the traits involved, such loci will result in a mixed population with strongly or weakly adaptive phenotypes that provide a subset of cells with a survival advantage within a given niche. Hypermutable loci in Nme are typically phase-variable loci, (**Figure 1**) in which the expansion and contraction of simple sequence repeats (SSR) result in stochastic expression of a trait within a population of bacterial daughter cells derived from a single progenitor. Hypervariable loci are typically loci in that contain both conserved functional regions and variable regions which contain variable epitopes that misdirect the host immune response (De Ste Croix et al., 2020). Such hypervariable regions are derived from contingency loci, some of which are partial and silent (such as the *pilS* cassettes), and some of which exist as multiple intact loci in the bacterial chromosome (such as the Opa-encoding loci) (Nassif et al., 1993; Callaghan et al., 2006). Both hypervariable and hypermutable loci are considered mechanisms of “short-sighted” evolution, typically driving *in host* evolution during colonisation and IMD (Green et al., 2020).

Despite the abundance of both hypervariable and hypermutable loci in Nme, multiple studies which compared hyperinvasive and commensal lineages have not detected an association between the phasome (the entire cohort of loci containing SSRs) and hyperinvasiveness (Wanford et al., 2018; Wanford et al., 2020; Mullally et al., 2021). The study by Mullally et al. (2021) proposed a model in which acquisition and loss of genomic islands correlated with the propensity of a lineage to cause IMD. Although the majority of genomic islands were hypothetical, where functions were known, they conferred traits associated with survival in host cells (e.g. resistance to host killing mechanisms) and competitive colonisation traits (e.g. bacteriocins and fratricidal competition mechanisms). In contrast to the hyperinvasive lineages, the commensal lineage cc53 possessed only 33 of the 93 genomic islands found in the pangenome. Interestingly, cc11 was found to be an outlier in this scheme, possessing the largest number of genomic islands (48/93) and by far the highest D/C ratio, suggesting that this lineage may be uniquely adapted to a pathogenic lifestyle (Mullally et al., 2021).

One possible explanation for these observations is the theory of coincidental evolution, in which virulence factors arise as a result of environmental selection pressures not directly associated with causing disease in the host per se (Sun et al., 2018). In this model, the first bottleneck encountered by Nme is colonisation of host mucosal surfaces and the need to out-compete the established microbiome. An epicellular lifestyle in which the bacteria invade the epithelial host cells, replicate and re-cycle to the apical surface has a dual purpose: to avoid competition from the microbiome but also to subvert nutritional immunity and evade host innate immunity. In this context, the accidental acquisition of the ability to cause IMD may potentially be an outcome of acquiring traits to improve bacterial growth for further transmission. One hypothetical pathway by which this could be achieved is the stimulation of inflammation and subsequent dysregulation of nutritional immunity, especially in the form of high lactate production. Conversely, stimulation of the inflammatory cascade results in the activation of adaptive immune responses, and in these circumstances Nme would need to develop resistance to host adaptive immunity mechanisms in order to take advantage of this carbon source. Although there are limited studies comparing genetic lineages and their ability to cause inflammation, a meta-analysis of the current published works in (**Table 1**) suggest that there are trends present to support this hypothesis. Typically, commensal strains of Nme (such as cc53) or strains isolated from carriage are less inflammatory than isolates from hyperinvasive lineages. Of the hyperinvasive lineages, cc11 has the strongest ability to stimulate inflammatory markers in whole blood, DCs, and epithelial cells, and induce increased host cell apoptosis compared to GGI and GGII isolates (Unkmeir et al., 2002a; Plant et al., 2006; Deghmane et al., 2009; Deghmane et al., 2011; Michea et al., 2013; Potmesil et al., 2014; Besbes et al., 2015; Aass et al., 2018) (**Table 1**). The capacity to induce increased levels of apoptosis and inflammation compared to other lineages is associated with the acquisition of multiple unique genetic islands, including NadA, and the possession of virulence-associated genomic islands associated with both GGI and GGII. In addition, the recent adaption of cc11 to the human urinogenital tract and subsequent capability to cause epidemic outbreaks of urethritis provides an exciting opportunity to examine this hypothesis in real time (Tzeng et al., 2017). In this case, adaptation to the urogenital niche included the loss of capsule production and the acquisition of anaerobic metabolism by genetic transfer from *N. gonorrhoeae* to enable improved colonisation and growth, respectively, in the human urogenital tracts of men. As further work is performed on this new pathotype, comparisons of inflammatory potential may inform further thoughts on how Nme has evolved in the past.

**Table 1:** Inflammation and immune modulation caused by *N. meningitidis* from different lineages.

Strain	Model	Cytokine/Chemokine expression	Cell surface marker expression/phenotype	Apoptosis	Overall inflammatory Profile	Reference
**cc11**	Epithelial cells		-TNF-RI, Sustained JNK activation, ↓NF-κB	↑	Highly inflammatory	(Deghmane et al., 2009; Deghmane et al., 2011; Besbes et al., 2015)
	Whole blood	↑TNF-α, IL-6, IL-10	Oxidative Burst ↑TLR2, TLR4, HLA-DR, CD14			(Potmesil et al., 2014; Aass et al., 2018)
	Dendritic cells	↑IFN-α, TFN-α, IL-6, IL-8	↑CD86			(Unkmeir et al., 2002a; Michea et al., 2013)
	Mouse sepsis model	↑IL-6, TNF-α, KC				(Plant et al., 2006)
**Invasive GGI isolates (cc5, cc60, cc22, cc23)**	Epithelial cells	↑TNF-α, IL-6, IL-8, IFN, IL-1β		↑	Inflammatory, low number of studies	(Guo et al., 2020)
	Dendritic cells	↑IL-6, IL-8, TNF-α				(Unkmeir et al., 2002a)
**Invasive GGII isolates (cc213, cc461, cc32, cc269, cc41/44)**	Epithelial cells	↑TNF-α, IL-6, IL-8, IFN, IL-1β		↓	Inflammation, reduced apoptosis	(Pridmore et al., 2001; Massari et al., 2003; Deghmane et al., 2009)
	Endothelial cells	↑IL-8, IL-6, RANTES, IFN-γ, CXCL8, CXCL1, CXCL2, CCL20				(Dick et al., 2017; Martins Gomes et al., 2019)
	Whole blood	↑TNF-α, IL-6, IL-10, IL-1β, IL-8	Oxidative Burst ↑TLR2, TLR4, HLA-DR, CD14			(Potmesil et al., 2014; Aass et al., 2018)
	Dendritic cells	↑IL-6, IL-8, ↑TNF-α, IFN-γ, IL-1β, GM-CSF	No induction of CD86, CD40, CD83, HLA-I, HLA-DR by live bacteria			(Clements et al., 2001; Unkmeir et al., 2002a; Kurzai et al., 2005; Jones et al., 2007)
	Macrophages	↑TNF-α, IL-6, IL-12, IL-10		↓		(Pridmore et al., 2001; Peiser et al., 2002; Tunbridge et al., 2006; Peak et al., 2016)
	Mouse	↑KC, MIP-1α, MIP-2, TNF-α, IL-1β				(Johswich et al., 2013)
	Meningeal cells	↑CXCL3, CXCL8, IL-8, IL-6, RANTES, MCP-1, TNF-α, IκBζ, G-CSF, GM-CSF, adrenomedullin				(Christodoulides et al., 2002; Borkowski et al., 2014)
	Neutrophils			↓		(Peak et al., 2016)
**Carriage strains (varying ST)**	Epithelial cells		↓TNF-RI, sustained NF-κB activation	↓	Supressed differentiation in immune cells, reduced apoptosis and inflammatory cytokine production	(Deghmane et al., 2009; Deghmane et al., 2011; Besbes et al., 2015)
	Meningeal cells	↑adrenomedullin reduced CXCL3, CXCL8, IL-8, TNF-α, IκBζ, G-CSF, GM-CSF				(Borkowski et al., 2014)
	Whole Blood	↑TNF-α, IL-6, IL-10	Oxidative Burst Reduced TLR2, TLR4, CD14			(Potmesil et al., 2014)
**IMD**	Patient CSF	↑IL-1α, IL-1β, IL-1ra, IL-2, IL-6, IL-8, IL-10, RANTES, G-CSF, GM-CSF, IFN-γ, TNF-α, MCP-1, MIP-1α, MIP-1β, MIF ↑C1q, MBL, C3a, iC3b, C5a, sC5b-9, CFH			↑	(Mook-Kanamori et al., 2014; Johswich, 2017)

While relatively little experimental biological work has been done on cc53, there is evidence of a distinct strategy of co-existence with the host. These isolates lack a capsule and the broad protection against complement, antibody-mediated opsonisation, and phagocytosis that provides. They lack many of the other features common to the hyperinvasive lineages including the Opc invasin, the HpuAB system for iron acquisition from heme, the MDA phage, *O*-acetylated pilin glycans and an IgA1 protease capable of cleaving IgG3. cc53 and other carriage-restricted lineages are less inflammatory and induce reduced cytokine production, apoptosis, and differentiation of a range of immune cells, indicating an overall strategy of persistence within the nasopharynx in a similar manner to the commensal *Neisseria* species.

## Conclusions

Nme has proven to be an exciting model for understanding the evolution of epicellular bacterial colonisation in humans. The remarkable plasticity of meningococcal genome has allowed this species to develop both commensal and pathogenic lifestyles in multiple host niches. Future work on understanding the interference between Nme and the human microbiome, how Nme interacts with the epithelial surface at a molecular level, and how these processes differ between genetic lineages will enable a greater understanding of commensalism and virulence of *Neisseria* spp.

## Author Contributions

AM and NM contributed equally to research and drafting of the manuscript. AM, NM, and CK contributed to editing the manuscript. All authors contributed to the article and approved the submitted version.

## Conflict of Interest

The authors declare that the research was conducted in the absence of any commercial or financial relationships that could be construed as a potential conflict of interest.

## Publisher’s Note

All claims expressed in this article are solely those of the authors and do not necessarily represent those of their affiliated organizations, or those of the publisher, the editors and the reviewers. Any product that may be evaluated in this article, or claim that may be made by its manufacturer, is not guaranteed or endorsed by the publisher.
